# A single-agent extension of the SIR model describes the impact of mobility restrictions on the COVID-19 epidemic

**DOI:** 10.1038/s41598-021-03721-x

**Published:** 2021-12-28

**Authors:** Matteo Paoluzzi, Nicoletta Gnan, Francesca Grassi, Marco Salvetti, Nicola Vanacore, Andrea Crisanti

**Affiliations:** 1 Departament de Física de la Matèria Condensada, Universitat de Barcelona, C. Martí Franquès 1, 08028 Barcelona, Spain; 2 CNR-ISC, Institute for Complex Systems UOS “Sapienza”, Piazzale A. Moro 2, 00185 Rome, Italy; 3 Department of Physics, Sapienza University of Rome, Rome, Italy; 4 Department of Physiology and Pharmacology, Sapienza University of Rome, Rome, Italy; 5 Department of Neurosciences, Mental Health and Sensory Organs, Sapienza University of Rome, Rome, Italy; 6 IRCCS Istituto Neurologico Mediterraneo Neuromed, Pozzilli, Italy; 7 National Center for Disease Prevention and Health Promotion, Istituto Superiore di Sanità, Rome, Italy

**Keywords:** Population dynamics, Computational science, Scientific data, Applied physics

## Abstract

Mobility restrictions are successfully used to contain the diffusion of epidemics. In this work we explore their effect on the epidemic growth by investigating an extension of the Susceptible-Infected-Removed (SIR) model in which individual mobility is taken into account. In the model individual agents move on a chessboard with a Lévy walk and, within each square, epidemic spreading follows the standard SIR model. These simple rules allow to reproduce the sub-exponential growth of the epidemic evolution observed during the Covid-19 epidemic waves in several countries and which cannot be captured by the standard SIR model. We show that we can tune the slowing-down of the epidemic spreading by changing the dynamics of the agents from Lévy to Brownian and we investigate how the interplay among different containment strategies mitigate the epidemic spreading. Finally we demonstrate that we can reproduce the epidemic evolution of the first and second COVID-19 waves in Italy using only 3 parameters, i.e , the infection rate, the removing rate, and the mobility in the country. We provide an estimate of the peak reduction due to imposed mobility restrictions, i. e., the so-called *flattening the curve* effect. Although based on few ingredients, the model captures the kinetic of the epidemic waves, returning mobility values that are consistent with a lock-down intervention during the first wave and milder limitations, associated to a weaker peak reduction, during the second wave.

## Introduction

People mobility and interactions are key events in epidemic spreading. Recent works, related or not to Covid-19 pandemics, have shown how travel distance and duration impacts on the diffusion of transmissible diseases^1,2^. In many studies, mobility data have been derived from mobile phone traffic changes^1–6^ and remarkably good fits of the observations have been obtained using compartmental models.

These models describe the spread of infectious diseases among homogeneous compartments with different health status. The basic model is the Susceptible-Infected-Removed (SIR^7^) model, in which the susceptible compartment evolves into Infected with an infection rate $\alpha$. The infected population will be then removed with a removing rate $\gamma$. To take into account the complexity of disease evolution, the basic SIR model has been generalized in several ways, introducing various sub-populations and describing, by different *reaction rates*, the conversion among interacting compartments. For instance, an Exposed compartment is added in SEIR models and Susceptible can become Exposed but not Infected. Exposed become Infected, and Infected evolve into Removed. In the case of Covid-19 outbreak, up to 8 population compartments have been used^8^.

Social interactions also influence disease spread and their inclusion in models of infectious disease spread is strongly advocated^9,10^. Simulations based on synthetic populations of hundreds thousands individuals can be performed (see^11^ as an example related to Covid-19). Alternatively, descriptions of average population behaviour can be performed, dividing people in age classes and identifying contact frequency and duration in the places normally attended by each age class (school, work, leisure, home etc.)^12,13^. Contacts within and among groups determine the values assigned to SIR reaction rates for each group. Reference values for these contact matrices have recently been proposed for the European population as a part of the POLYMOD project^14^ and used to forecast the spread of several pandemic outbreaks, such as avian influenza in 2008^14^ and Covid-19^15^. Values have also been recalculated during different phases of Covid-19 pandemic in some countries^16–18^ .

Even in the simplest form with only three populations, SIR models are very effective in describing epidemic spread^19^. The interplay between the two control parameters, $\alpha$ and $\gamma$, determines the evolution of the epidemic, with two possible scenarios for the evolution of the infection: in the first one, the epidemic wave spreads exponentially, reaches the epidemic peak, and then decreases. The second scenario deals with epidemic extinction: the new cases do not grow and the epidemic extinguishes exponentially fast. The basic reproduction number $R_t$, i. e., the average number of secondary infections caused by a primary case^20^, controls the crossover between these two regimes: when $R_t<1$, epidemic spreading ends. In the case of a SIR model $R_t$ can be computed analytically and its value is $R_t=\alpha / \gamma$. In the framework of SIR models, it is clear that there are at least two strategies for maintaining $R_t<1$. One can improve the capacity in removing infected subjects that corresponds to make $\gamma$ large, for instance thanks to pharmaceutical treatments that allow infected agents to heal or via non-pharmaceutical interventions (NPIs), such as isolation of suspected/ascertained cases. Alternatively, the value of the infection rate $\alpha$ can be reduced by vaccination or NPIs, such as physical distancing that modifies the structure of the social interactions. These mitigate or extinguish the epidemic spreading, as they change the social contact matrices and thus lower the probability of contagion. One of the effects of all NPIs is the so-called *flattening the curve* effect: The slowing-down of the epidemic spreading due to interventions makes the epidemic longer, as the attainment of herd immunity is delayed, but less severe because of an effective reduction of the epidemic peak which keeps infections within the limits of cases affordable by the healthcare system. In the first year of the current Covid-19 epidemic, NPIs were set in place in many countries, most often in the form of generalized lockdown, although in some cases prevention has relied on tracking and case isolation^21^. Following restrictions, in several countries the dynamics of infection growth changed from exponential to a power law^22^. This sub-exponential growth rate can be reproduced by a standard SIR models, introducing a new compartment that accounts for quarantined population^23^. However, being based on the dynamics of conversion between compartments, SIR models describe the average events in population groups, and cannot account for individual behaviours, which are relevant to the transmission of infectious diseases^9,10^. Indeed, a recent network-based analysis^24^ has shown that there is a critical number of interactions below which the number of infected people grows almost linearly. Interactions among people have been modelled using coarse-grained field theories such as dynamical density functional theory^25^. However, microscopic descriptions of individual agents bring details about epidemic spreading, with reference to several issues and have been used in studies about the spreading of infectious diseases for more than a decade^26^, also for Covid-19 pandemics. As mentioned above, very realistic society models are obtained by simulations involving tens of thousands individuals, each characterized by hundreds of parameters (for instance, see^11,27^). Of course, such a detail, although informative, becomes computationally very expensive. In this work, we have looked for a compromise between detail and simplicity, using methods typical of statistical mechanics. Social interactions in different environments (work, school, leisure places etc.), which determine infection spread, are ultimately modulated by people mobility. Depending on the degree of the details to reproduce, diverse approaches are suitable for taking into account the complexity of people mobility^28,29^. For instance, long-distance displacements by airplane travel have been superimposed to a standard SIR model in the form of additional stochastic equations that describe the dynamics of each compartment^12,29^. This approach is very detailed in terms of the destinations reached, but still relies on the homogeneous population assumption.

To be able to introduce individual behaviours in predicting how mobility restrictions, and hence reduction of social interactions, impact on epidemic spreading, we propose an approach that models mobility in a ”society” using single agents that move onto a two-dimensional grid. Within each cell of the grid agents interact according to a classical SIR. In order to mimic different ranges of human mobility, we assume that agents perform Lévy-walks, which are characterized by many short displacements, with occasional longer ones^30^. This movement pattern is used by animals belonging to many species across the evolutionary tree. It represents the foraging strategy of choice for rural human populations^31,32^ or for people playing in a virtual wild environment^33^, but also by human crowds approaching pedestrian crossings^34^. It has even been suggested that it is an imprinted strategy thanks to its evolutionary importance^31^. In non-rural settings, people normally alternate, long displacements to go to a place (for instance, from home to work), with short-range movements while they remain on the site for a while and Lévy walk well approximate this traveling pattern^30,34,35^. In this framework, mobility restrictions set in place to contain epidemic spread are modelled as limits imposed to the Lévy distribution of jumps, through a jump parameter. We show that the proposed model is able to account for the non-exponential epidemic growth induced by mobility restrictions and to reproduce real data from the first and the second COVID-19 epidemic wave in Italy, using a minimal set of parameters. The model also gives insight into the equilibrium between mobility reduction and case tracing/isolation and the possibility to exempt population groups from mobility limitations.

The main advantage of the proposed model is its flexibility and analytical power: by changing the density of agents and the jump parameter, different types of social interactions can be studied. Transmission and recovery rates can be adapted to other diseases. Finally, different levels of mobility restrictions can be introduced, adapting the model to different situations.

## Results

### Combining agent mobility patterns and SIR model

**Figure 1:**
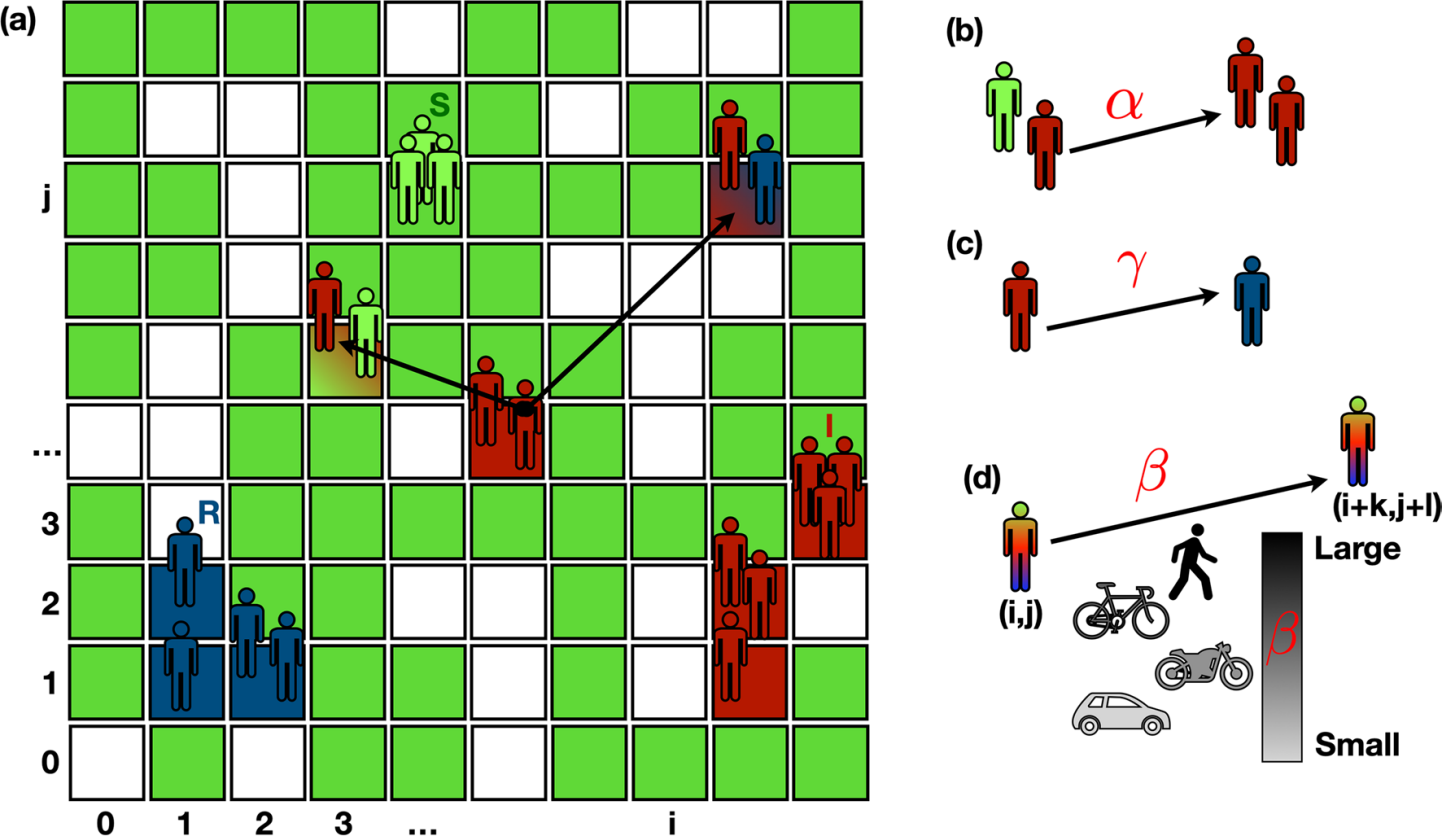
Agent-based SIR model on a lattice. **(a)** Agents of different colors, representing the SIR states, move on a lattice. White cells represent empty sites. Green cells are occupied by susceptible (S) agents, blue cells contain only removed (R) agents. Red cells contain only infected (I) agents. Shaded cells contain agents in a mixture of states. Agents can move among cells performing jumps (black arrows) whose length follows Lévy statistics. The letters *i* and *j*, with $i=1,..,N_b$ and $j=1,...,N_b$ define the location of the cell (*i*, *j*). **(b,c)** Agents in the same cell undergo a SIR dynamics: **(b)** S become I at a rate $\alpha$; **(c)** I become R at rate $\gamma$. **(d)** The jump dynamics allows an agent to move from the cell (*i*, *j*) to $(i+k,j+l)$. The probability to perform a large/small jump is controlled by the parameter $\beta \in [1.0,1.99]$. Large $\beta$ values correspond to small jumps, i. e., a random walk that gives rise to Brownian motion. Small $\beta$ values correspond to large jumps.

**Figure 2:**
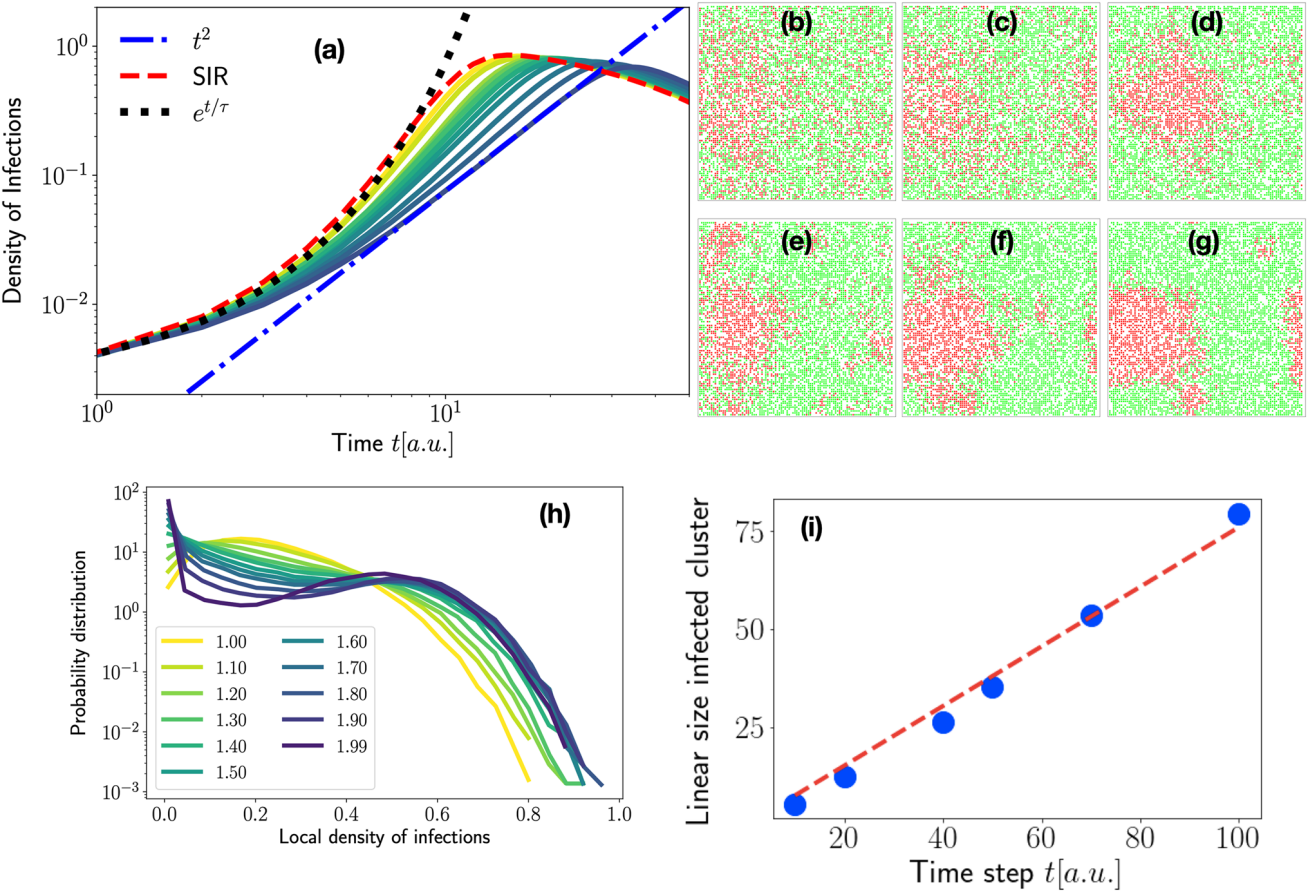
Agent dynamics impacts the epidemic spreading process. (**a**) The graph shows the dependency of the epidemic curves on $\beta =1.20,1.50,1.75,1.80,1.85,1.87,1.90,1.92,1.95,1.97,1.99$ (increasing values of $\beta$ from yellow to violet). As $\beta$ decreases, the epidemic grows exponentially fast (dotted black curve) and approaches the evolution of SIR model in well-mixed population (dashed red curve). The dash-dot blue curve is a power law $\sim t^2$. The parameters of the SIR reactions are $\alpha =0.9$ and $\gamma =0.025$. (**b**–**g**) Typical configurations taken at the same fraction of infected agents $I/N \sim 0.25$ for increasing values of $\beta =1.0,1.2,1.4,1.6,1.8,1.9$ (red are infected sites, green the susceptible ones, we keep white the sites populated by removed agents). (**h**) The probability distribution function of the local density of infected sites. (**i**) Radius of the cluster of infected agents ($\beta =1.99$) as a function of time. The red dashed line is a linear fit.

To take into account agent mobility^19^ in a scenario compatible with a SIR model, we developed the model pictorially illustrated in Fig. 1. As explained in details in the Methods Section, the agents can move on a lattice through jumps processes, modelled using a Lévy walk of jump parameter $\beta$^36–38^. When $\beta$ becomes large, i.e., for $\beta \rightarrow 2$, agents tend to perform a Brownian random walk with very short jumps. As $\beta \rightarrow 1$, agents can travel long distances in just one step. There are no constraints on the number of agents that can occupy a single cell. In each cells, agents can be infected by neighbours according to the SIR rules. Thus, the parameters that control the model are the jump parameter $\beta$ plus the standard SIR parameters, infection rate $\alpha$ and removal rate $\gamma$. The agent-based lattice model considered here reduces to a standard SIR model when the well-mixed population condition is satisfied, i. e. when large jumps dominate the dynamics (Fig. 2).

For reproducing the kinetics of real data we made the following assumptions:

- In the absence of containing strategies, the infection is characterized by a high infection rate (we take $\alpha =0.9$) and a low removal rate ($\gamma =0.025$ or 0.05). Using as a unit of time the update of all agent positions (see Methods for details), the removal rate introduce a time scale $\tau _I = \gamma ^{-1}=40$ or $20$. This characteristic time scale represents the average time an agent remains infected and can thus spread the infection. This condition ensures that we are in an epidemic regime, i. e., the mean-field value is $R_t \gg 1$. We stress that, since the SIR dynamics with only three sub-populations is a simplification of the real chain of epidemic transmission, the parameters we choose for the epidemic spreading are not strictly related to those of Covid-19. Because we are interested in the effect of mobility restriction on epidemic spreading, we fix the epidemic parameters in a way that, without mobility restrictions, we are sure to stay in the worst-case scenario with an exponentially fast spreading of the infection.
- The parameter $\beta \in [1,1.99]$ tunes the intensity of mobility restrictions. The higher its value, the stricter the limitations. $\beta$ is one of the fitting parameters.
- Other interventions that mitigate the epidemic spreading tend to increase the removal rate $\gamma$. We thus assume that $\gamma$ is another fitting parameter. This is because typical measures, for instance, quarantine, remove infected agents from the system. In this way, we reabsorb the presence of many hidden sub-populations into an effective value of $\gamma$.
- We define the parameter $\delta$, i. e., the fraction of infected agents at the epidemic peak with respect to the entire population, that provides a quantitative measure of the reduction of the epidemic peak. In other words, the parameter $\delta$ represents the efficiency of a given containing strategy compared to the uncontrolled situation where all the agents turn out to contract the infection (which is the case of our model for $\gamma \ll \alpha$, $\alpha =0.9$, and $\beta =1$).

To detail how mobility restrictions induce deviations from the SIR model, we calculate, via numerical simulations, the epidemic curves as a function of time for different values of $\beta$ as illustrated in Fig. 2a. Here, the SIR parameters are $\alpha =0.9$ and $\gamma =0.025$, i. e., the corresponding SIR model is in the fully blown epidemic regime. For small $\beta$ the epidemic growth is well captured by the exponential function, indicating that we are in the epidemic regime. As $\beta$ increases the curve turns out to be flattened and the peak reduces to $80\%$. Moreover, the growth of the epidemic for the largest $\beta$ examined is well described by the power law $I(t) \sim t^{2}$. The value of the exponent is comparable with those measured in different countries during the COVID$-19$ epidemic wave^23^. The model considered here suggests that the crossover from exponential growth to power-law might be related to changes of the mobility patterns that, in our picture, shift from being dominated by large jumps to small ones. This finding is consistent with the observation that a sub-exponential growth in the number of infected people is a consequence of containing strategies^23^. Moreover, in the microscopic description adopted here, the crossover in the kinetics of *I*(*t*) is driven by just one parameter.

The crossover from exponential to power-law growth reflects the drastic change in the structure of clusters of infected agents, as illustrated in Fig. 2b–g, where typical configurations with the same fraction of infected agents are shown ($I/N=0.25, \alpha =0.9, \gamma =0.025$). As one can see, in the high mobility region ($\beta = 1$), infected agents are spread almost everywhere in the system. As $\beta$ increases, infected sites tend to form a single cluster. This phenomenology is consistent with the literature of mobile agents undergoing SIR dynamics^39,40^. This structural change is quantitatively documented by the density distribution of infected sites shown in panel (h) of the same figure (see section Methods for details). As one can appreciate, the distribution becomes double-peaked as $\beta$ increases. The first peak around zero indicates the presence of an extended region of susceptible agents. The peak at high values is due to the growing cluster of infected agents. As highlighted in panel (i), the cluster grows linearly in time and thus the number of infected grows with $t^2$.

**Figure 3:**
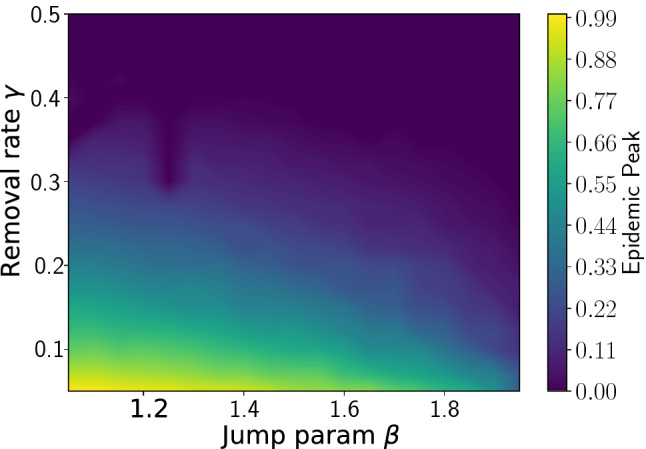
Effect of different containment strategies. The phase diagram is obtained considering as control parameters $\beta$, that represents mobility restrictions, and $\gamma$, the efficiency in removing infected agents. The color scale represents the fraction of the initial susceptible population that becomes infected, ranging between 0 (epidemic suppression, violet region) and 1 (fully-blown epidemic, yellow region). Containment is achieved as $\beta$ increases (corresponding to increasing mobility restrictions) even with low removal rate, or increasing $\gamma$ (effective removal of infected agents), even with limited mobility restrictions.

Another interesting aspect to understand with this model is the trade off between mobility restrictions and and other kind of interventions that have the effect of increasing the removal rate. In particular in Asian countries^41^, NPIs applied during the COVID-19 waves have relied mostly on contact tracing and/or preventive quarantine, with little mobility reduction, leading to effective and durable control of epidemic spreading, as reviewed by Ref.^21^. To understand if there is an optimal balance between containing strategies (characterized by $\beta$) and efficiency in removing infected agents (denoted by $\gamma$), we calculate the fraction of infected population at the epidemic peak (the maximum of *I*(*t*)) as a function of the jump parameter $\beta$ and of the removal rate $\gamma$. As above, the initial occupation number of each site is, on average, one. The infection rate is $\alpha =0.9$. The resulting phase diagram is shown in Fig. 3. The color indicates the fraction of infected population: in the violet region, this fraction goes to zero (epidemic is suppressed) while in the yellow region such a value goes to one, indicating an epidemic regime. The phase diagram fully recapitulates the effectiveness of the two strategies used to mitigate the infection spread, a strong lockdown with limited contact tracing, or an efficient contact tracing a moderate reduction of the mobility.

**Figure 4:**
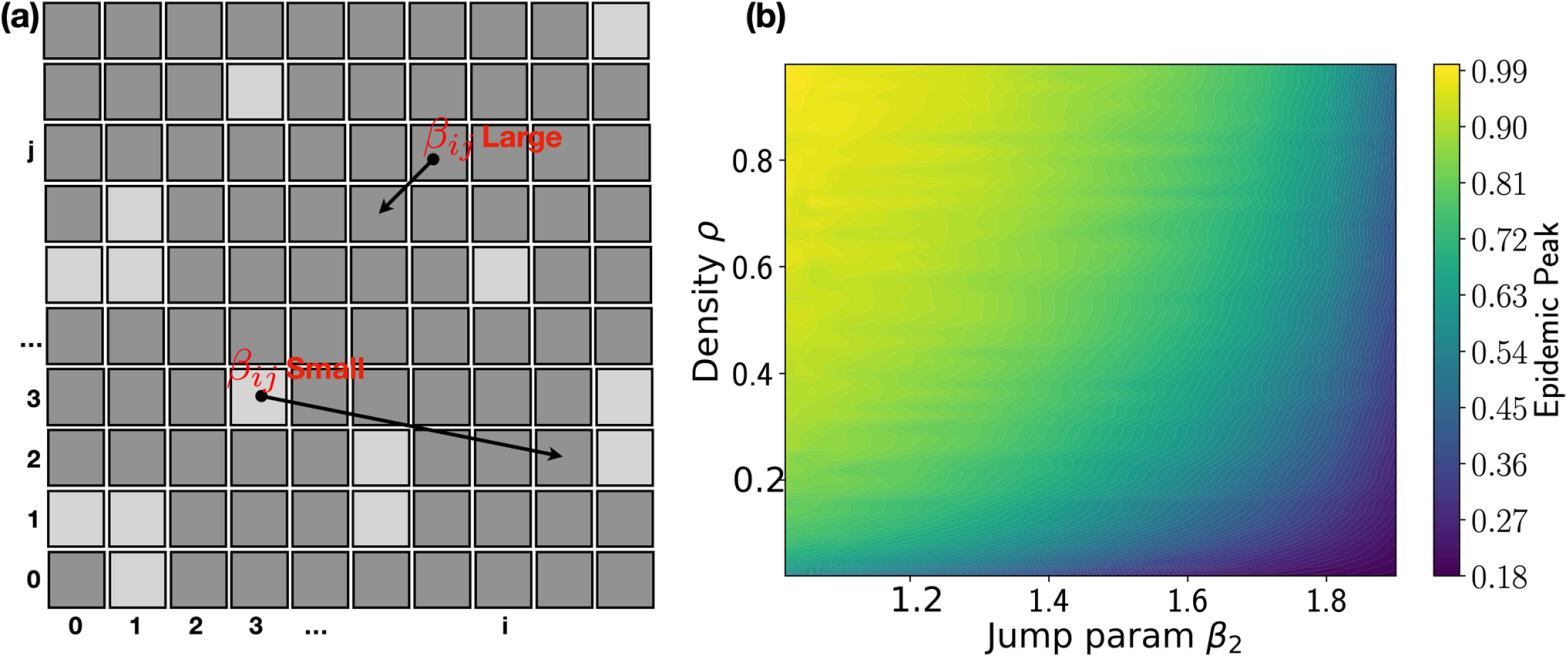
Sites of different mobility affect epidemic spreading. (**a**) Each cell labelled by (*i*, *j*) is characterized by its own mobility parameter $\beta _{ij}$. We consider the special case of a binary mixture ($\beta _{ij} = \beta _{1,2}$) of high and low mobility regions. Changing the density $\rho$ of $\beta _2$ sites and the value of $\beta _2$, we obtain the the phase diagram presented in panel (**b**), obtained for $\beta _1=1.99$, $\alpha =0.9$, and $\gamma =0.05$, conditions that grant contained epidemic spreading thanks to the low-mobility group. A small amount of sites with small values of $\beta _2$ can trigger the epidemic spreading.

However, even under the strictest lockdown, several activities could not be stopped (hospitals, food supply chain, ...), meaning that a single mobility parameter cannot fully describe this varied situation. To understand what could be the impact of heterogeneous motility patterns on the evolution of the epidemic, we introduce in the model some regions characterized by a high mobility (jump parameter, $\beta _2$), while the majority of the the cells have restricted mobility, with a jump parameter $\beta _1=1.99$ (see Methods for more details). By varying $\beta _2$ and the density of more mobile cells (parameter $\rho$) we are able to draw the phase diagram shown in Fig. 4.

As in the previous case, in the violet area the epidemic spreading is stopped, while in the yellow area the epidemic peak reaches the entire population. Epidemic spreading takes place above a critical curve: for a given value of mobility $\beta _2<\beta _1$, the system can support a maximum fraction of regions with that mobility. Above that fraction, the system falls into an epidemic regime. It turns out that even a small fraction of regions with $\beta _2<1.9$ triggers the epidemic spreading. Our results confirm that, in the absence of contact tracing able to mitigate the spreading of the infection, only those activities that are strictly necessary should be carried on in order to prevent an enhancement of infections.

### The first and second COVID-19 epidemic wave in Italy

**Figure 5:**
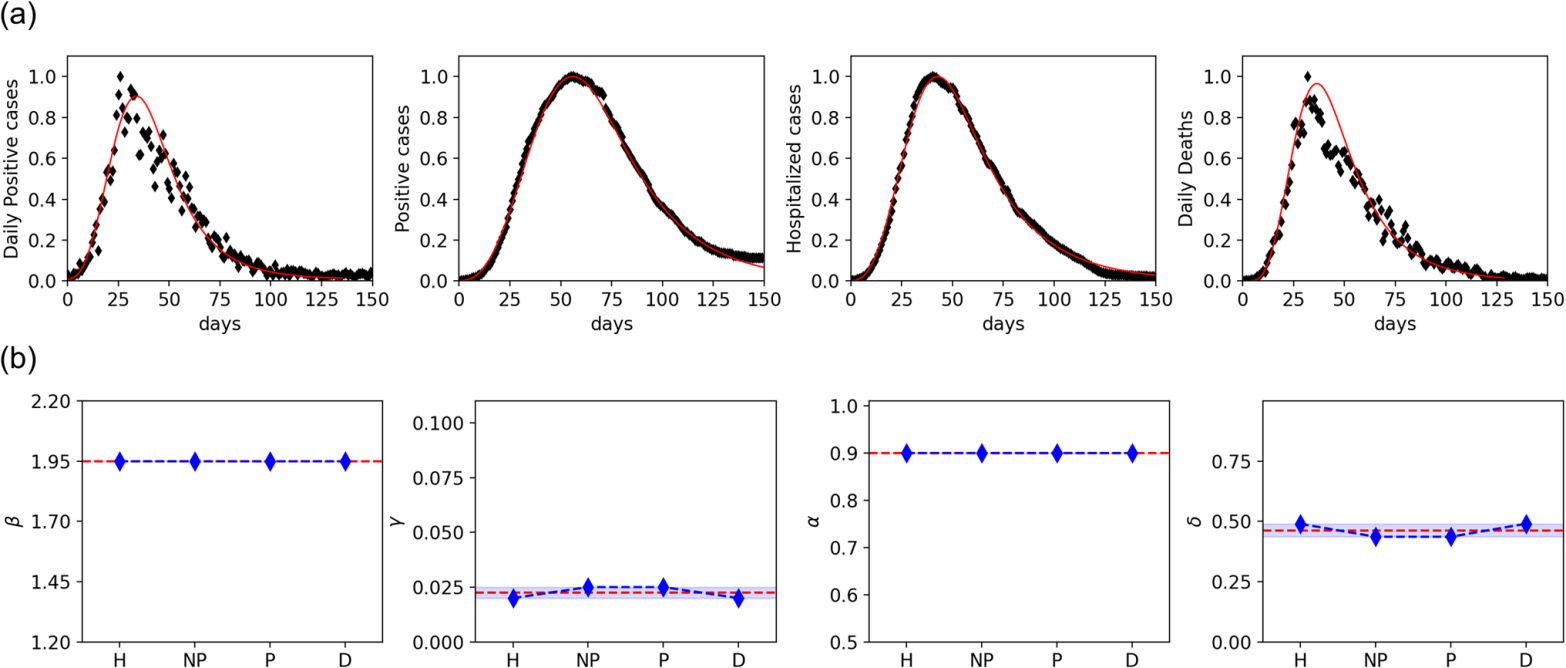
Model description of the first epidemic wave. **(a)** Time evolution of the epidemic curves in Italy during the first 150 days for Daily positive cases (NP), Positive cases (P), Hospitalized cases (H) and Deaths (D), as indicated. Black symbols are data, red curves are the fits to the model. Data (from^42^) are normalized at the peak value. Time is measured in days from the day zero, February 4th, 2020. **(b)** Mobility parameter $\beta$, removal rate $\gamma$, infection rate $\alpha$ and the corresponding peak reduction $\delta$ are indicated for the four indexes considered in **(a)**. The shaded area represents the standard deviation, dashed red line is the average.

As the proposed model is able to describe the transition from an exponential to a power-law epidemic growth, and is capable to provide meaningful predictions on the epidemic trend using only few parameters, it is important to validate it with real data. Therefore, we next tested the model against data from the first and second waves of COVID-19 in Italy. The epidemiological data are very sensitive to the ability of a given country in testing the population, identifying new cases among asymptomatic people, which reflects the capacity of the health system^2^. During the first wave, testing was restricted by lack of reagents, so the number of positive individuals has been largely underestimated. Daily data for the second wave are much more reliable, thanks to more extensive testing. For this reason, we look at the time evolution of different indexes that are (i) Daily new positive cases (NP), (ii) Positive cases at a given time (P), (iii) Hospitalized cases (H), and (iv) Daily deaths (D). The latter two indicators are independent of testing capacity. The fitting parameters are the mobility parameter $\beta$, and the removing rate $\gamma$, while a quantitative measure of the *flattening the curve* effect, is given by the parameter $\delta$. The infection rate is kept fixed to the value $\alpha = 0.9$ for all the simulations. As discussed above, $\delta$ represents the fraction of agents that do not contract the infection. The mobility parameter $\beta$ and the removing rate $\gamma$ can be tuned to reproduce the epidemic spreading. We use official data released daily from the Presidency of the Council of Ministers - Department of Civil Protection^42^. As shown in Fig. 5 there is a very good agreement between data and model predictions. The model appears to fit particularly well the number of hospitalized cases (H) and the total number of positive cases (P). Both indexes are time-accumulated data, known to lead to an underestimation of uncertainty of fits^43^. However, daily number of new cases (NP) and deaths (D) are also well fit, and the four indexes considered provide estimates for the fitting parameters $\beta$, $\gamma$ and $\delta$ that are consistent with each other, supporting the validity of the model. Thus, the observed evolution of the indexes is well captured by the model with $\beta = 1.95$, meaning strong mobility restrictions, and $\gamma \sim 0.025$. The resulting peak reduction ($\delta$) is around $50 \%$.

**Figure 6:**
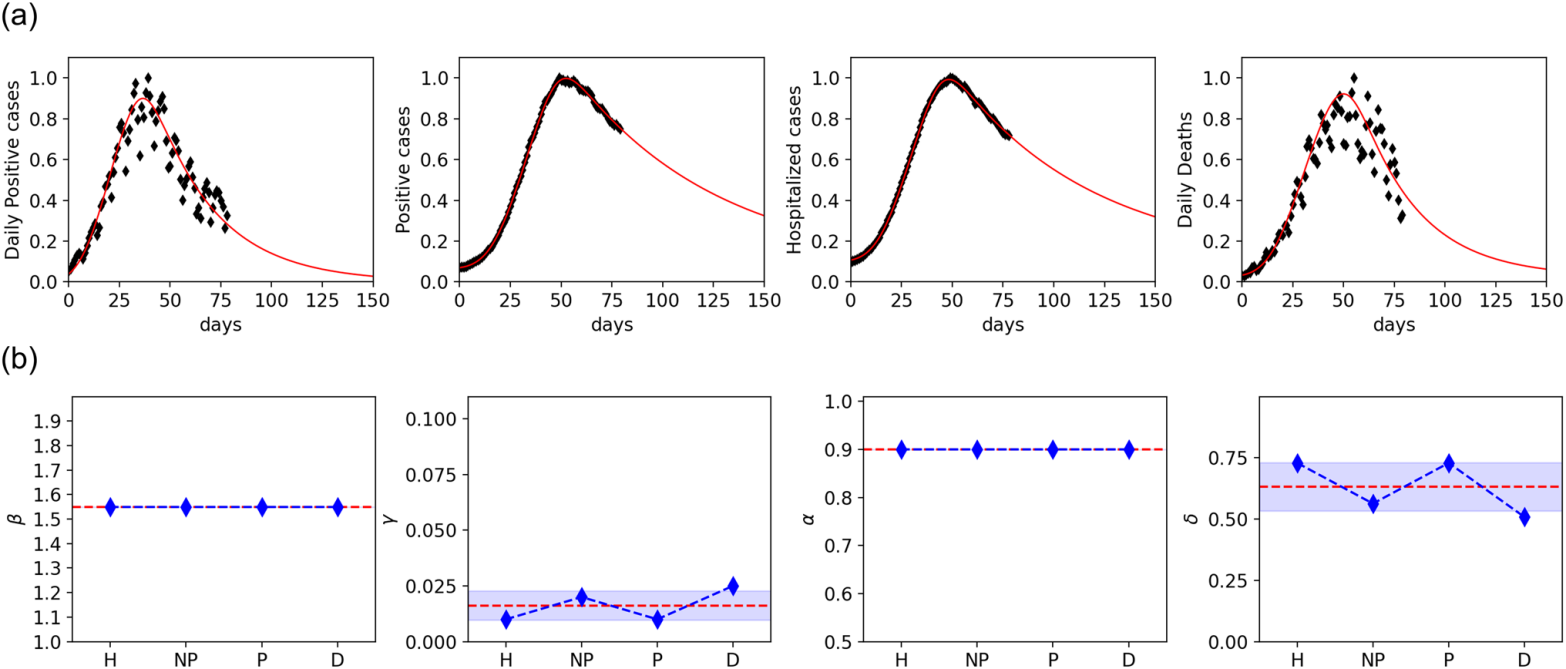
Model description of the second epidemic wave in Italy. **(a)** Time evolution during the first 75 days of the second wave of Daily positive cases (NP), Positive cases (P), Hospitalized cases (H) and Deaths (D), as indicated. Black symbols are data, red curves are the fits to the model. Data (from^42^) are normalized at the peak value. Time is measured in days from the day zero, September 20th, 2020. **(b)** Mobility parameter $\beta$, removal rate $\gamma$, infection rate $\alpha$ and the corresponding peak reduction $\delta$ are indicated for the four indexes considered in **(a)**. The shaded area represents the standard deviation, dashed red line is the average.

For a further validation of our model, we perform a comparison between the model and the data of the second epidemic wave. As a starting date, we have taken September 20th, and data are updated to December 28th, 2020. For fitting, we have subtracted the baseline and normalized values to the epidemic peak. Results are shown in Fig. 6. As for the first wave, fit to daily (NP and D) and cumulative data (H and P) yield comparable values of the three parameters, $\beta$, $\gamma$ and $\delta$. The second wave is described by a lower value of $\beta = 1.55$, as a consequence of less severe restrictions, which were less effective, as shown by a smaller reduction of the epidemic peak, described by $\delta \sim 72.5 \%$. Note that, also for the fit of the second wave, the infection rate has been kept fixed to $\alpha =0.9$ while the values of $\gamma$ found in the two waves are almost the same, indicating that the main difference in the growth of the epidemic can be attributed to a different mobility during the two waves. The values of $\gamma$ and $\alpha$ we obtain from the fit are almost the same during the two waves. This fact indicates that $\beta$ turns out to be the significant fitting parameter. Although at a very coarse-grained level, in the sense that the parameters of the model are not sensitive to the details of the mobility restriction imposed during the two epidemic waves, the model captures the efficiency of the lock-down on March 2020 and correctly returns a small value of $\beta$ with a substantial peak reduction. The second wave, characterized by mobility restriction heterogeneous in space and time, i. e., different implementations in different regions, are reflected by a higher value of $\beta$ that suggests the presence of regions of different mobility. Moreover, the strong relationship between peak reduction and mobility restriction provides clear evidence of the crucial role played by NPI in containing the epidemic.

## Discussion and conclusions

The SIR model has been introduced almost a century ago^7^ to describe the spread of infectious diseases among population compartments with different health status and used successfully to model real epidemics. Being based on the dynamics of conversion between compartments, the model describes the average behaviour of population groups. Recently, it has been also extended to account for mobility (see for instance Ref.^1^), but still with reference to population compartments. To be able to describe individual behaviours while retaining simplicity and analytical power of the SIR scheme, we have used methods typical of statistical mechanics and introduced a single-agent extension of the SIR model where mobility is taken into account. Some considerations on the strengths of the model can be done.

*(i) The model is simple since it uses only three parameters, the infection rate* ($\alpha$), *the removing rate* ($\gamma$), *and the mobility* ($\beta$). Agents move in jumps on a grid, with a dynamics based on Lévy walks of exponent $\beta$ for exploring different regions of the grid. NPIs based on mobility restrictions can be thus modeled as increasing the parameter $\beta$ towards the limit $\beta \rightarrow 2$, which corresponds to Brownian walks^36^. Since for $\beta \rightarrow 1$ the model approaches the limit of a SIR in a well-mixed population, we can provide an estimate of the peak reduction, i. e., the *flattening the curve effect* due to mobility limitations. The model shows that, in principle, mobility reduction can not only induce the *flattening the curve effect*, but can also trigger the epidemic extinction. However, close to the crossover between epidemic spreading and epidemic extinction (see Fig. 3), for a given ability in removing (or healing) the infected agents, a small change in mobility might affect dramatically the epidemic evolution. Our model suggests that strategies based only on mobility reduction are not suitable for containing the epidemic spreading since a small amount of high mobility agents can trigger epidemic waves (as shown in Fig. 4).

*(ii) The model is computationally affordable and can be implemented without a detailed design of society structure, at variance with more sophisticated agent-based models.* To describe data from the COVID-19 epidemics we have done some assumptions. The first one is that mobility is homogeneous in space and thus $\beta$ is not space-varying, and the second assumption is that the complicated structure of sub-populations can be reabsorbed into an effective value of the removing rate $\gamma$. These choices have allowed us to maintain a small number of fitting parameters.

*(iii) The model is effective, as it correctly recapitulates the different restrictions to mobility imposed in Italy during the first and second waves.* As a first step, we have looked at global data integrated over the country, using the assumptions described above. In this picture, mobility restrictions in Italy contributed to reduce the peak of the epidemic by about $50\%$ during the first wave, and by about $25\%$, during the second wave. Combining both epidemiological and mobility data^2^, it has been shown that, in the framework of a metacommunity Susceptible-Exposed-Infected-Recovered (SEIR) model, emergency containment measures reduced the transmission by $45 \%$ during the first epidemic wave in Italy, that is compatible with our results. We emphasize that, in our picture, mobility is described by the parameter $\beta$. This means that we can extract useful information for monitoring the effects of mobility restrictions on the pandemic wave. For instance, we can estimate the actual degree of mobility limitations consequent to the imposed restriction, or the peak reduction with respect to the worst-case scenario. If the fitted value of $\beta$ is small, it means that —*effectively*— the mobility has not been reduced. On the other hand, larger values of $\beta$ indicate that prescribed limitations have been actually implemented. The estimates of $\beta$ are compatible with the different levels of mobility restrictions set in place during the two waves. The $\beta \rightarrow 2$ values during the first wave might be considered compatible with a lock-down situation. On the other hand, during the second wave, interventions have been heterogeneous in the country with regions of higher/lower mobility. This scenario might be compatible with, on average, a smaller $\beta$ value and, as a consequence, less efficient control of the peak reduction. It is interesting to note that, even in its simplest form with a single value of the $\beta$ parameter, the proposed model adequately represents the nation-wide evolution of the epidemic wave. Likely, this occurs because mobility restrictions dominate the NPIs set in place. Finally, we notice that our estimate of $\gamma$ does not change during the two waves, i. e., $\gamma \sim 0.02$. Since $\gamma \ll \alpha$, there is not epidemic extinction. However, we have shown that combining mobility reduction with interventions that increase the removing rate $\gamma$, it is possible to trigger an epidemic extinction even in the case $\gamma < \alpha$.

*(iv) The model is versatile, as it can be used to estimate the effectiveness of other interventions, or different mobility rates.* As shown by the examples discussed here, from its simple form, the model can be adapted to include several sub-populations or regions with different $\alpha$, $\beta$ and $\gamma$ values (for instance to mimic young and old people) or to include spatial heterogeneity, in which different interventions are applied. Moreover, the model might be also extended to include fluxes of agents. In this way, it would be possible to study how the injection of infected mobile agents can trigger epidemic waves.

Given the above observations, we can state that the proposed model is flexible and reliable thus offering the potentiality to be exploited in future works on social interactions or transmission of diseases.

## Methods

### Model with homogeneous restrictions

**Figure 7:**
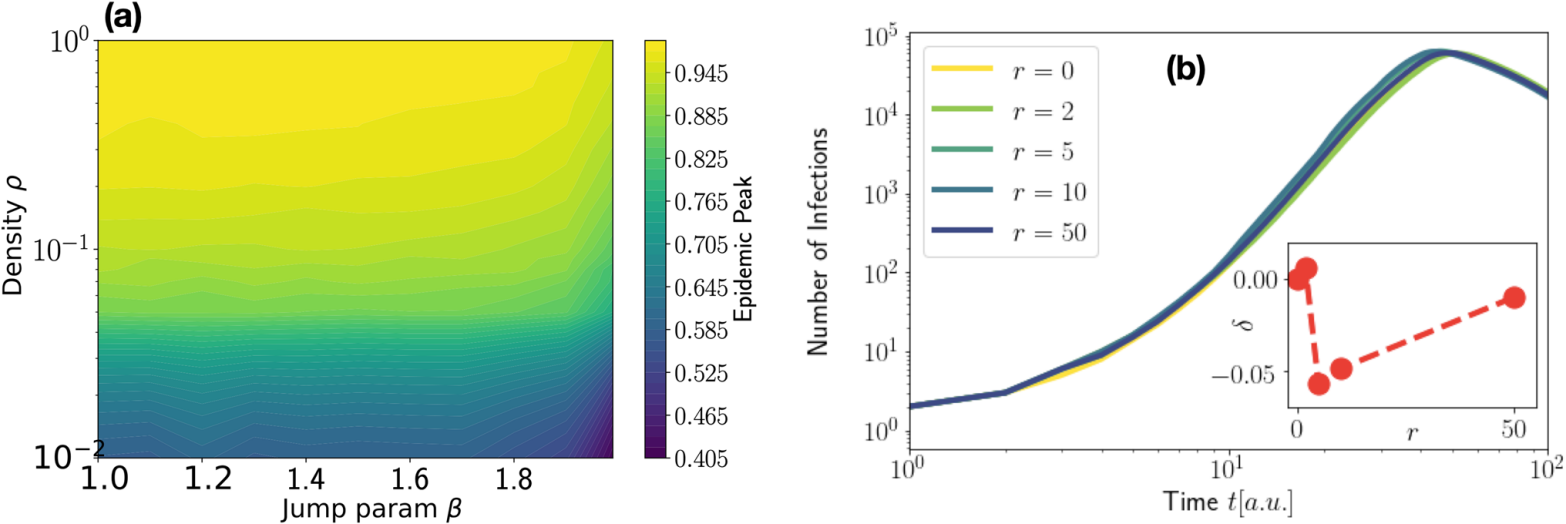
Effect of average density and position resetting. (**a**) The phase diagram has been obtained by varying the average density ${\bar{\rho }}$ and the parameter $\beta$ with $\alpha =0.9$ and $\gamma =0.05$. (**b**) Number of infections for $\alpha =0.9$, $\gamma =0.025$, and $\beta =1.2$ for different values of resetting time, i. e., $r=0,2,5,10,50$ (in time-step unit). Inset: peak reduction as a function of *r*.

The model consists of *N* point-like agents that can move on a two dimensional lattice of $N_c \times N_c$ cells with periodic boundary conditions. The side of the simulation box is $L=300$ and the linear size of each cell of the lattice has been set to $\ell = L / N_c = 1$. At the beginning of the simulations, the agents cover uniformly the lattice with an average density ${\bar{\rho }}=N/L^2$. We have considered different average densities ranging from ${\bar{\rho }}=1$ to ${\bar{\rho }}=0.01$. As it is shown in Fig. 7, where we report the phase diagram obtained by varying $\beta$ and ${\bar{\rho }}$ with $\gamma =0.05$ and $\alpha =0.9$, the average density ${\bar{\rho }}$ has to be reduced by an order of magnitude for obtaining a substantial reduction of the epidemic spreading. It is worth noting that the model does not take into account some aspects of human everyday life, as for instance the fact that people usually visit some preferred places more than others and interact more often with specific groups of people. We can argue that, in our framework, the overall effect of these preferential interactions is to slow down the spreading of the infection. To prove this we have performed additional simulations in which, after some time steps *r*, the positions of all the agents are reset to their initial value. Results are shown in Fig. 7b for different reset times. As one can appreciate, the resetting mechanism has a very little effect on the epidemic spreading, as documented in the inset of the same panel where we show the peak reduction $\delta$ as a function of *r*.

To perform the data fits of the COVID-19 pandemic, we have used simulations with ${\bar{\rho }}=0.6$. We do not employ any restriction on the number of agents that can stay at the same time step in a given cell. Interactions occur only among agents within the same cell. As a standard procedure^19^, each agent brings a state variable that describes the SIR state, i. e., Susceptible (*S*), Infected (*I*), and Removed (*R*). For contracting the infection, a susceptible (*S*) agent has to occupy a cell where there is at least one infected (*I*) agent, as sketched in Fig. 1. The infection is contracted by a susceptible agent with a rate $\alpha$ (for each Infected agent presents in the cell). Infected agents (*I*) are removed (*R*) with rate $\gamma$ i. e., an infected agent remains infectious for an average time $\tau _{I} = \gamma ^{-1}$. The dynamics is implemented as follows. For each agent labelled by *i*, with $i=1,..,N$, we propose a displacement $\Delta \mathbf{r}_i= r_i (\cos \theta _i, \sin \theta _i)$, with $\theta _i$ a random angle extracted by a uniform distribution, i. e., $\theta _i \in [0,\pi ]$, and $r_i$ extracted by a Lévy distribution of parameter $\beta$. The Lévy statistics is obtained using the Mantegna’s algorithm^44^. Because of the periodic boundary conditions, we choose to bound $r_i$ between 0 and *L*/4. Random numbers have been generated using the standard C library. The simulation is organized as follows: *N* susceptible agents are randomly distributed among cells, one of them is randomly selected as a seed of the infection changing its state from *S* to *I*. We first update the SIR state of each agent in each cell and then we update the position of each agent. A complete updating of all the agent positions fixes the unit of time. Moreover, the position of each agent is updated at every step. The curves *I*(*t*) have been obtained by averaging over $N_s=100$ independent runs. Each run starts with one infected agent. The probability distribution function of local density has been computed by dividing the system into cells of linear size $\ell _c=5$ and counting the number of infected lattice sites on each cell. The local density has been computed on configurations containing the same fraction of infections, i. e., $I/N\sim 1/4$. For computing the distribution function, we have performed averages on 200 independent configurations.

We then tested the model ($I_m(t)$) against data ($I_d(t)$). To this aim, we performed several simulations with different $\gamma$ and $\beta$ values, while keeping $\alpha =0.9$ and we established which set of parameters provide the ${\tilde{I}}_m(t)$ curve that best match ${\tilde{I}}_d(t)$ where the upper tilde indicates that both curves have been normalized to their own maximum. Best curves are obtained by minimizing the distance $d[{\tilde{I}}_{d},{\tilde{I}}_{m}]=\int _{t_{start}}^{t_{max}} dt \, | {\tilde{I}}_{d}(t) - \lambda {\tilde{I}}_{m}(\kappa t+t_{start},\alpha ,\beta ,\gamma )|^2 \;,$ with $t_{start} \in [0,5]$. Most of the fits have been performed with $\lambda =1.0$ but in case of noisy data such as for the Daily Positive Cases this value has been varied in the interval $\lambda \in [0.9,1.0]$ to obtain a better match.

### Effects of non-homogeneous mobility restrictions

The case in which some population groups retained almost normal mobility while the majority of the population was restricted, was inserted in our model considering a binary mixture of regions described by a jump parameter $\beta _1$ or $\beta _2$, as sketched in Fig. 4. The regions are chosen randomly at the beginning of the simulation, introducing the density $\rho$ of the sites having jump parameter $\beta _2$. We started from a situation of epidemic extinction, using $\beta _1=1.99$ (agents perform small jumps), $\alpha =0.9$, and $\gamma =0.05$. We then changed $\beta _2$ from 1.05 to 1.99 and the density of $\beta _2$ sites $\rho \in [0,1]$.
